# SARS-CoV-2 identified by transmission electron microscopy in lymphoproliferative and ischaemic intestinal lesions of COVID-19 patients with acute abdominal pain: two case reports

**DOI:** 10.1186/s12876-021-01905-3

**Published:** 2021-08-26

**Authors:** Albert Martin-Cardona, Josep Lloreta Trull, Raquel Albero-González, Marta Paraira Beser, Xavier Andújar, Pablo Ruiz-Ramirez, Jaume Tur-Martínez, Carme Ferrer, José Angel De Marcos Izquierdo, Anna Pérez-Madrigal, Laura Goiburú González, Jorge Espinós Perez, Maria Esteve

**Affiliations:** 1 Department of Gastroenterology, Hospital Universitari Mútua Terrassa, Universitat de Barcelona, Terrassa, Barcelona, Spain; 2 Centro de Investigación Biomédica en Red de Enfermedades Hepáticas y Digestivas (CIBEREHD), Madrid, Spain; 3 Department of Pathology, Hospital del Mar, Universitat Autònoma de Barcelona, Barcelona, Spain; 4 Department of Pathology, Hospital Universitari Mútua Terrassa, Universitat de Barcelona, Terrassa, Barcelona, Spain; 5 Department of Radiology, Hospital Universitari Mútua Terrassa, Universitat de Barcelona, Terrassa, Barcelona, Spain; 6 Department of Surgery, Hospital Universitari Mútua Terrassa, Universitat de Barcelona, Terrassa, Barcelona, Spain; 7 Intensive Care Unit, Hospital Universitari Mútua Terrassa, Universitat de Barcelona, Terrassa, Barcelona, Spain

**Keywords:** SARS-CoV-2, COVID-19, Intestinal lymphoma, Ischaemic colitis, Transmission electron microscopy, Case report

## Abstract

**Background:**

SARS-CoV-2 may produce intestinal symptoms that are generally mild, with a small percentage of patients developing more severe symptoms. The involvement of SARS-CoV-2 in the physiopathology of bowel damage is poorly known. Transmission electron microscopy (TEM) is a useful tool that provides an understanding of SARS-CoV-2 invasiveness, replication and dissemination in body cells but information outside the respiratory tract is very limited. We report two cases of severe intestinal complications (intestinal lymphoma and ischaemic colitis) in which the presence of SARS-CoV-2 in intestinal tissue was confirmed by TEM. These are the first two cases reported in the literature of persistence of SARS-CoV-2 demonstrated by TEM in intestinal tissue after COVID 19 recovery and SARS-CoV-2 nasopharyngeal clearance.

**Case presentation:**

During the first pandemic peak (1st March–30th April 2020) 932 patients were admitted in Hospital Universitari Mútua Terrassa due to COVID-19, 41 (4.4%) required cross-sectional imaging techniques to assess severe abdominal pain and six of them (0.64%) required surgical resection. SARS-CoV-2 in bowel tissue was demonstrated by TEM in two of these patients. The first case presented as an ileocaecal inflammatory mass which turned to be a B-cell lymphoma. Viral particles were found in the cytoplasm of endothelial cells of damaged mucosa. In situ hybridization was negative in tumour cells, thus ruling out an oncogenic role for the virus. SARS-CoV-2 remained in intestinal tissue 6 months after nasopharyngeal clearance, suggesting latent infection. The second patient had a severe ischaemic colitis with perforation and SARS-CoV-2 was also identified in endothelial cells.

**Conclusions:**

Severe intestinal complications associated with COVID-19 are uncommon. SARS-CoV-2 was identified by TEM in two cases, suggesting a causal role in bowel damage.

## Background

SARS-CoV-2, the aetiological agent of coronavirus disease 2019 (COVID-19), like many viruses with respiratory involvement, can cause gastrointestinal symptoms. In the first series, 5% presented nausea, vomiting, and diarrhoea [1]. More recent cohorts and meta-analysis showed that diarrhoea and digestive symptoms are much more frequent, ranging from 8.0 to 61% [2, 3]. Different mechanisms of diarrhoea induced by SARS-CoV-2 have been proposed. One of them is viral invasion of the gastrointestinal mucosal cells through attachment to the angiotensin-converting enzyme 2 (ACE2) [3–5]. This receptor is present in gastric mucosal cells, in the enterocytes of the small intestine and colon. It was found colocalizing with SARS-CoV-2 by confocal microscopy, proving that this virus can invade almost the entire gastrointestinal tract [6]. The infective capacity of SARS-CoV-2 of the enterocyte has been demonstrated, by using transmission electron microscopy (TEM), in human small intestinal organoids [7] and in tissue samples of one patient with rectal cancer that was operated during the incubation period [8]. Apart from that, few cases of COVID-19 with severe intestinal complications have been reported [9]. The majority of them occurred in the setting of multiorgan failure without having the certainty of to what extent the virus had a direct involvement in the physiopathology of inflammation.

TEM allows direct visualization of SARS-CoV-2 in tissues of COVID-19 patients and provides good understanding of coronavirus replication in cells. However, the demonstration of SARS-CoV-2 by TEM outside the respiratory tract has been scarcely reported [10, 11] and evidences are still lacking in tissues from COVID-19 patients.

We report two patients with COVID-19 presenting with intestinal complications (one of them with an intestinal lymphoma and the other developing an ischaemic colitis) in which the presence of SARS-CoV-2 in endothelial cells of the intestinal tissue was detected by TEM. In addition, we demonstrated, for the first time, that SARS-CoV-2 remained in a latent status after COVID-19 recovery by analysing sequential intestinal samples in a 6-month period. The potential oncogenic role of SARS-CoV-2 was investigated through In situ hybridization (ISH) for viral sequences in lymphoma cells.

**Fig. 1:**
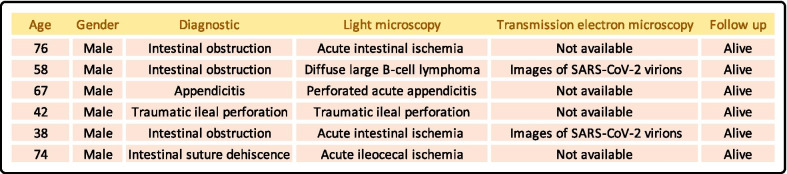
COVID-19 patients operated during the first pandemic peak (from March 1 to April 30, 2020). This figure shows the diagnoses, histological and transmission electron microscopic findings of patients operated during the first peak of the pandemic in our hospital

In order to ascertain how often severe intestinal complications occurred in COVID-19 patients we retrospectively reviewed the clinical records of the 932 patients admitted to our hospital during the first pandemic peak (March 1 to April 30, 2020). During this period, sixty abdominal cross-sectional imaging studies due to acute abdominal pain were performed in 41 out of 932 (4.4%) patients (mean age 68 years ± 13.5 SD, 63.4% men). Six of them (0.64%) required surgical resection due to perforation or obstruction, including the two patients described herein in whom SARS-CoV-2 involvement in the bowel damage was suspected (Fig. 1).

## Cases presentation

The first case was a 58-year-old male admitted on March 19, 2020, due to abdominal pain. He had mild hypertension and dyslipemia. On physical examination, he was haemodynamically stable, with 38.5ºC fever and with a hard mass in the right iliac fossa without peritonitis. Blood tests revealed haemoglobin 11.2 g/dL, normal glucose, platelet and leucocyte counts, normal renal function﻿, C-reactive protein (CRP) 43.3 mg/L, international normalized ratio (INR) 1.3, and D-dimer 1460 ng/mL. Basal arterial blood gas (21%) showed the following values: pH 7.41, pCO^2^ 38 mmHg, pO^2^ 71 mmHg, HCO^3^ 24.1 mmHg, Sat. O^2^ 94.2%.

**Fig. 2:**
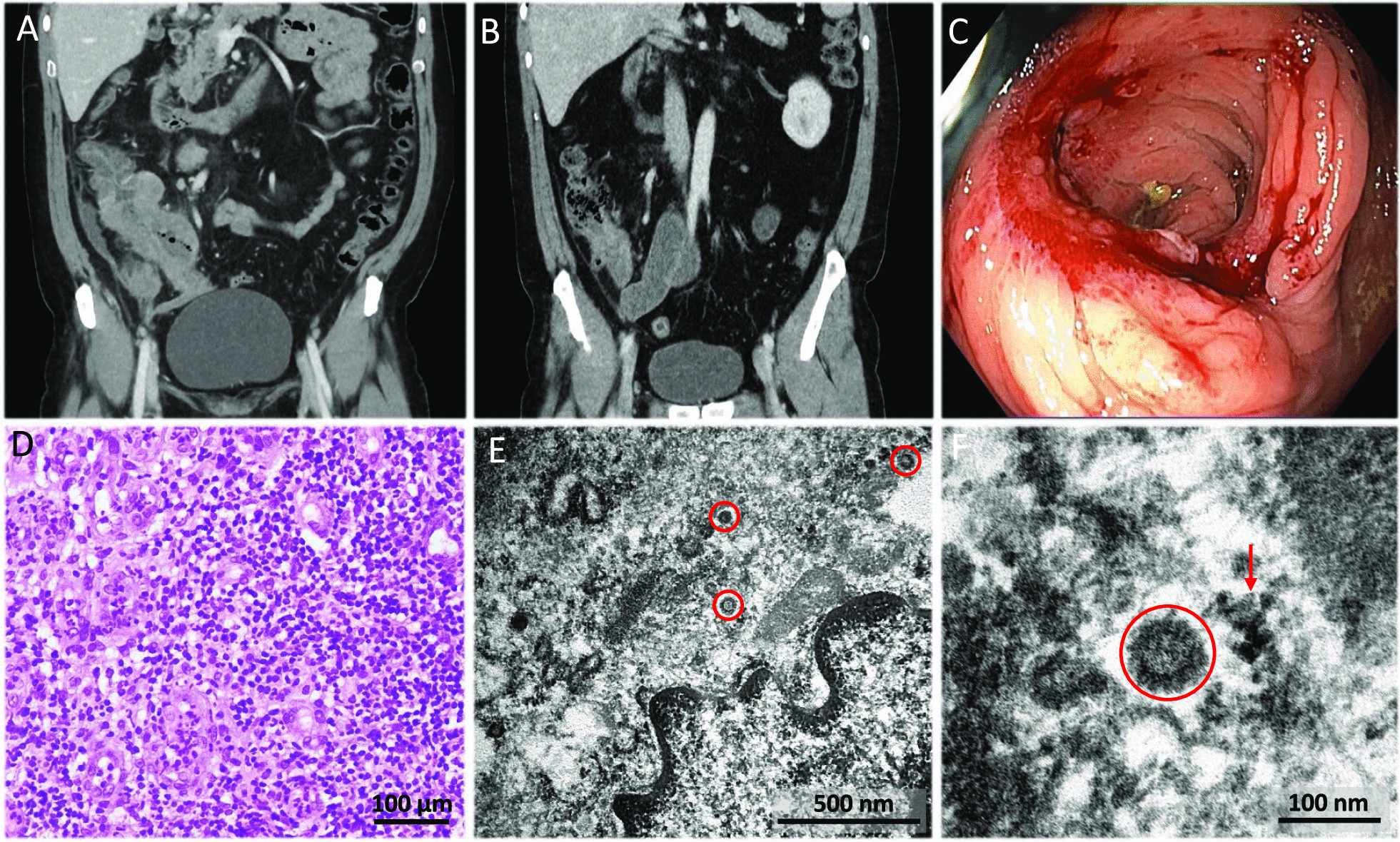
Complementary examinations (CT scan, colonoscopy, light microscopy and transmission electron microscopy) from case 1. **A**, **B** Coronal contrast-enhanced computed tomography (CT) scan at the moment of diagnosis (**A**) and after COVID-19 treatment (**B**). Panel **A** shows luminal narrowing and marked wall thickening involving the ascending colon, caecum, and terminal ileum. Panel B shows significant decrease in the wall thickening of the colon and terminal ileum, the lymph nodes have also decreased in size and number. **C** Colonoscopy at the level of the caecum reveals a mass of inflammatory appearance that predominantly affects the ileocecal valve, which is stenosed and prevents passage to the terminal ileum. **D** Haematoxylin and eosin staining of colonic biopsies showing abundant granulation tissue with lymphoplasmacytic inflammation and vascular proliferation (×200). **E** Electron micrograph of a portion of an endothelial cell showing several viral particles (red circles) lying apparently free in the cytoplasm, all separate from each other. **F** Close-up electron micrograph of one of the viral particles in **E**. The virus surface protrusions (red circle) are distinguished from confounding structures, such as ribosomes in tangential sections of the rough endoplasmic reticulum (red arrow), by the more geometric, lighter, hollow-looking appearance of the former

An abdominal computed tomography (CT) scan showed thickened walls in the ascending colon and caecum extending to the terminal ileum and appendix that suggested a neoformative process. Several enlarged lymphadenopathies were also found (Fig. 2A). A positron emission tomography (PET) scan was performed with a staging intention, which described an intensely hypermetabolic mass with standard uptake value (SUV) max31 in the caecum, and hypermetabolic lymphadenopathies in the mesenteric area. In addition, PET scan reported a poorly defined pulmonary uptake area in the right upper and middle lobes and in the left apical region (SUVmax7.7). Chest X-ray showed local patchy shadowing in the right middle lobe. All these pulmonary findings were suggestive of SARS-CoV-2 pneumonia, which was confirmed by a nasopharyngeal smear polymerase chain reaction (PCR) test. Treatment with oxygen therapy (high-flow mask, venturi type at 28% at 5 litres) and pharmacological treatment for COVID-19 were instituted with hydroxychloroquine 200 mg/12 h orally (PO), ceftriaxone 1 g/24 h intravenous (IV), azithromycin 500 mg/24 h PO and Lopinavir/Ritonavir 400 mg/100 mg/12 h PO. The treatment schedule did not include any corticosteroid agents at any time during hospital admission. The patient underwent clinical recovery, disappearance of fever, frank reduction of the abdominal mass (Fig. 2B), blood test normalization and pulmonary infiltrate improvement. After discharge, a colonoscopy was performed on April 15, 2020. An inflammatory mass occupying most of the caecal circumference was found, and the ileocecal valve was stenotic, preventing passage to the ileum (Fig. 2C). Multiple biopsies of the ascending colon were taken, showing, on light microscopy, abundant granulation tissue with lymphoplasmocytic inflammatory infiltrates and macrophages without granuloma formation. Immunohistochemical staining ruled out the presence of either an infiltrative epithelial neoplasm or a lymphoproliferative process (Fig. 2D). Cytomegalovirus (CMV) immunostaining was negative. PCR performed on formalin-fixed/paraffin-embedded (FFPE) tissue to detect atypical mycobacterial infection and SARS-CoV-2 were also negative.

The rapid improvement in parallel with COVID-19 recovery raised the suspicion of intestinal inflammation induced by SARS-CoV-2. In order to demonstrate this, TEM of the colon tissue, retrieved from the FFPE block, was performed. On ultrastructural study, viral particles characteristic of coronavirus were found. They showed the typical outer surface with several spikes all around them; rarely were more than ten found per cell, and they were well apart from each other. They were found in the cytoplasm of endothelial cells, from capillaries and arterioles in the involved mucosa. An electron-lucid area around some of these particles made them quite visible at low power. They were usually situated in the centre of an irregular and apparently empty space resembling rough endoplasmic reticulum. This ‘halo’ apparently prevented direct contact of the viral particles with cytoplasmic organelles (Fig. 2E, F).

**Fig. 3:**
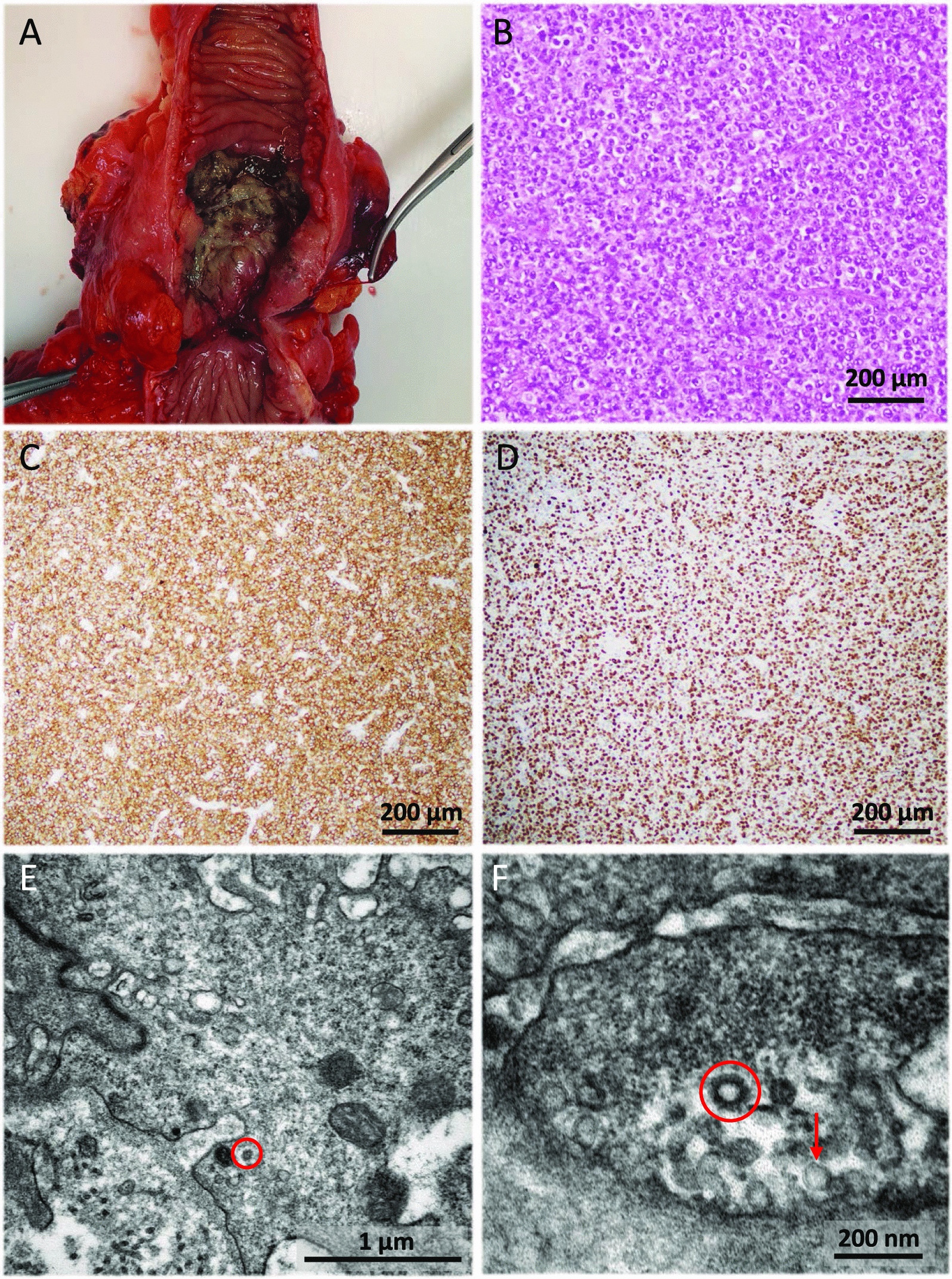
Complementary examinations (intestinal resection specimen, light microscopy and transmission electron microscopy) from case 1. **A** The ileocaecal resection specimen showed an ulcerated lesion with fibrosis of the intestinal wall, causing stenosis. **B** Haematoxylin and eosin staining of the surgical specimen showing diffuse large B-cell lymphoma germinal center subtype. Lymphocytes of medium and large size with irregular nuclei, vesicular chromatin, conspicuous nucleoli and mitotic figures were observed (×100). **C**- **D** Immunohistochemistry of the lesion was performed, showing expression for CD20, CD79a, bcl2, CD10, bcl6, LMO2, MUM1, and c-MYC. The tumour was classified as diffuse large B-cell lymphoma (DLBCL) germinal center subtype. In panel C, CD20 (clone L26, Ventana, Roche, Tucson, AZ, USA) immunohistochemistry is shown (×100). In panel **D**, a high proliferative index (Ki67) (clone 30-9, Ventana, Roche, Tucson, AZ, USA) was observed (90% in the hotspot areas) (100x). **E**- **F** Electron micrograph of the lymphoma tissue, in which viral particles are highlighted (red circles). **E** Viral particles (red circle) remained in occasional endothelial cells. **F** In contrast to coronavirus particles (red circle), pinocytotic vesicles (red arrow) have a smooth contour, their cell membrane lipid bilayer may be visualized, and they are often arranged in clusters

**Fig. 4:**
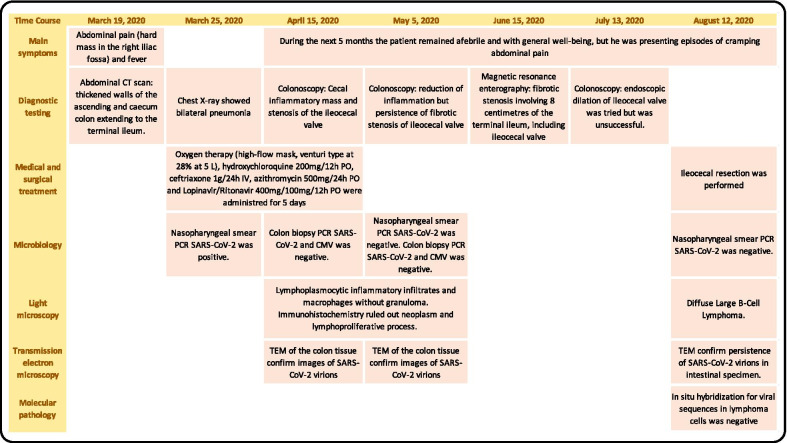
Time course of the patient's 1 (A) main events. This figure shows the main events and complications of the case 1. The first row of the table shows the dates of the events, and the first column describes the complementary examinations carried out

During the next 3 months the patient remained afebrile and in overall good condition, but he presented self-limited episodes of abdominal pain. Two more CT scans and colonoscopies showed progressive decrease in the size of the inflammatory mass and of the lymph nodes, but persistence of predominantly fibrotic stenosis of the terminal ileum including the ileocecal valve. Ileocaecal resection was indicated that was performed on August 12, 2020 (Fig. 3A). Microscopic examination of a surgical specimen showed infiltration of the caecum and ileum by a neoplastic lesion with a diffuse growth pattern, formed by atypical, medium to large-sized lymphocytes, with scant cytoplasm, irregular nuclei, vesicular chromatin and conspicuous nucleoli. These morphological features were suggestive of lymphoma. Immunohistochemistry study was performed, showing expression for B-Cell markers (CD20, CD79a), as well as expression for bcl2, CD10, bcl6, LMO2, CD21, MUM1, and c-MYC in the neoplastic cells. Staining for CD23 ruled out the presence of a dendritic cell network. A high proliferative index (Ki67) was observed (90% in the hotspot areas). ISH technique for Epstein-Barr virus was negative. The tumour was classified as a diffuse large B-cell lymphoma (DLBCL), germinal center subtype (according to Hans’ algorithm) (Fig. 3B–D). No component of low-grade lymphoma was identified. Rearrangements of *MYC*, *BCL2* and *BCL6* genes were studied by FISH, detecting translocations in *BCL2* and *BCL6*, but not in *MYC.* TEM of the lymphoma showed very scanty but convincing inclusions. In the endothelial cells of the lymphoma blood vessels, after very careful study, particles with coronavirus morphology were detected (Fig. 3E, F). Finally, an RNA in situ hybridization (RNA-ISH) assay of lymphoma and endothelial cells with SARS-CoV-2 was performed with negative results (Fig. 4).

**Fig. 5:**
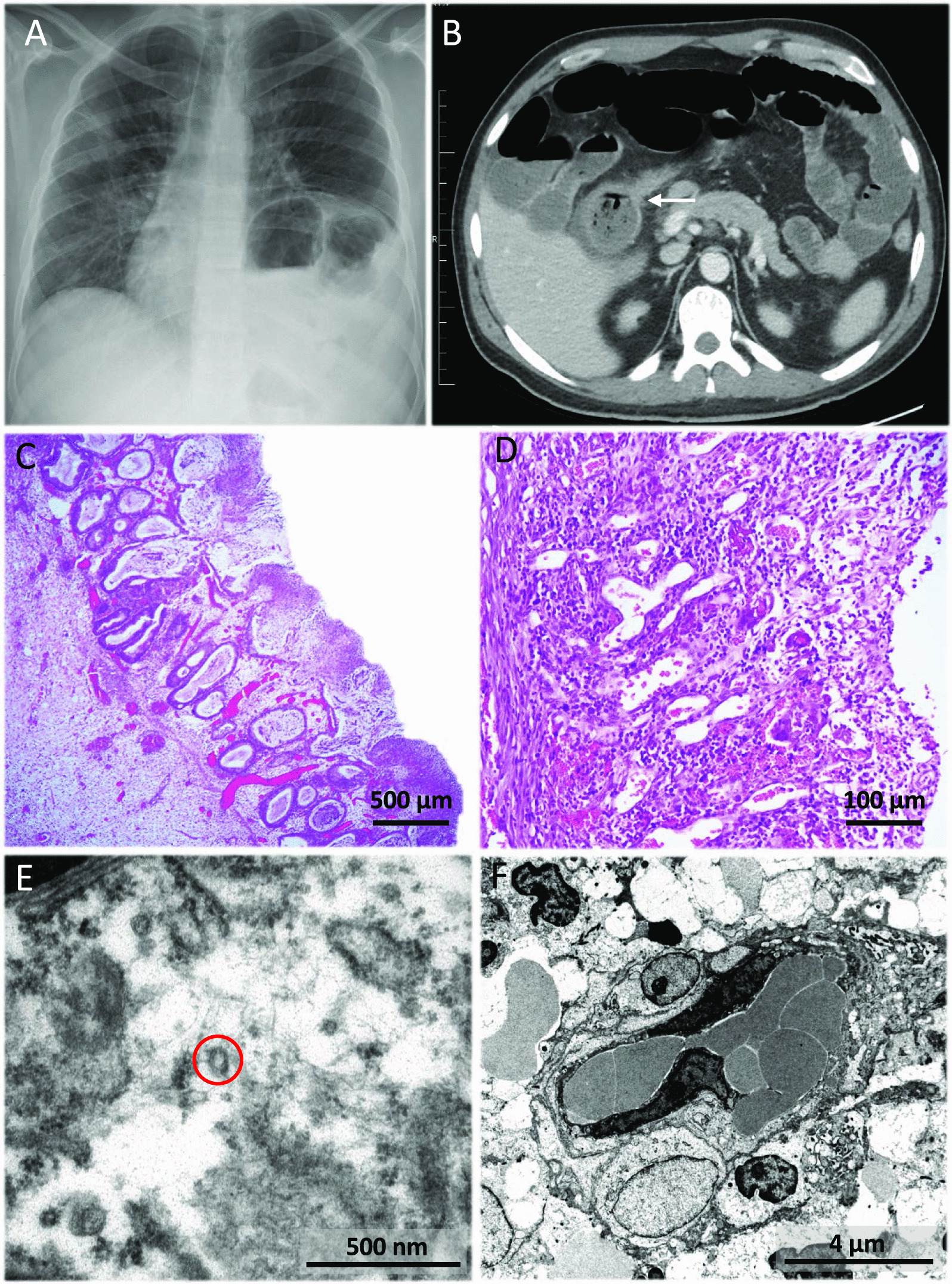
Complementary examinations (PA Chest X-ray, CT-scan, light microscopy and transmission electron microscopy) from case 2. **A** PA chest radiograph shows ground-glass opacification of bilateral perihilar region and the peripheral middle third of the right hemithorax**. B** Abdominal contrast-enhanced CT showing a short stenosis of the proximal transverse colon with mild involvement of the pericolic fat and proximal dilation of the ascending colon and small intestine (white arrow). **C**-**D** Haematoxylin and eosin staining of the surgical specimen. **C** Mucosal necrosis, haemorrhage, and submucosal oedema were observed (×40). **D** Transmural ulcer showed abundant granulation tissue, chronic inflammation, fibrosis, and steatonecrosis, findings consistent with intestinal ischaemia (×200). **E** Electron micrograph of the surgical specimen showing virus particles (red circle) in an oedematous, damaged endothelial cell. **F** Low-power high-resolution electron micrograph with markedly oedematous endothelial cells and pericytes, congested capillaries with red blood cells and platelets, endothelial cell containing enlarged nuclei and active nucleoli. All these features are a reflection of endothelial cell stress, damage, and reactive changes

The second case was a 38-year-old male patient, without relevant medical history, was admitted on March 18, 2020 for fever, dry cough, dyspnoea, and non-bloody diarrhoea. On physical examination he was haemodynamically stable and he had fever (39.2ºC) and bilateral rhoncus. Initial blood analysis showed normal haemogram, normal glucose, proteins, renal and liver function, INR 1.2, and D-Dimer 217 ng/mL. Arterial blood gas analysis performed with a high-flow mask (50% venturi type at 10 litres) showed pH 7.45, pCO^2^ 21 mmHg, pO^2^ 95 mmHg, HCO^3^ 19.7 mmHg, and Sat.O^2^ 97%. Chest X-ray showed right peripheral patchy shadowing due to COVID-19 (Fig. 5A) that was confirmed by positive SARS-CoV-2 nasopharyngeal PCR.

Treatment was started with ceftriaxone 1 g/24 h IV, azithromycin 500 mg/24 h PO, lopinavir/ritonavir 400 mg/100 mg/12 h PO, hydroxychloroquine 200 mg/12 h PO, Interferonβ1b 0.25 mg/mL subcutaneously every 48 h and dexamethasone 20 mg/24 IV. The patient was transferred to the intensive care unit (ICU) due to acute respiratory failure for orotracheal intubation and norepinephrine perfusion. He developed acute renal failure with oliguria of multifactorial cause (hypotension, diarrhoea, and rhabdomyolysis). After 1-month, mechanical ventilation was withdrawn and he was transferred to a conventional ward where oral diet was restarted with good tolerance. Control chest X-ray showed almost complete disappearance of alveolar infiltrates. On May 6, 2020, the patient suddenly presented abdominal pain, predominantly in the right hemiabdomen with signs of peritonitis, diaphoresis, and fever (38.5ºC). His status worsened, with haemodynamic instability, and respiratory and renal failure. An abdominal CT scan was performed, showing a stenosis in the transverse colon and a marked thickening of the right colon wall, surrounded by fat stranding (Fig. 5B). An emergency right hemicolectomy with terminal ileostomy was performed. Gross examination showed the colonic mucosa to have a ground cobblestone appearance, focal ulceration, and deposition of fibrinoid material. Histologically, mucosal necrosis, submucosal oedema, and haemorrhage were seen. The transmural ulcer showed granulation tissue, chronic inflammation, fibrosis, and steatonecrosis consistent with intestinal ischaemia. The adjacent mucosa showed regenerative changes, distorted architectural pattern, and mucinous hyperplasia. There was no evidence of CMV infection, nor of an infiltrative lesion (Fig. 5C, D). PCR for *Clostridium difficile* (CD) was positive in colon samples. A TEM of the colon tissue was performed, showing viral particles with a morphologic distribution essentially similar to those of case 1 (Fig. 5E, F).

**Fig. 6:**
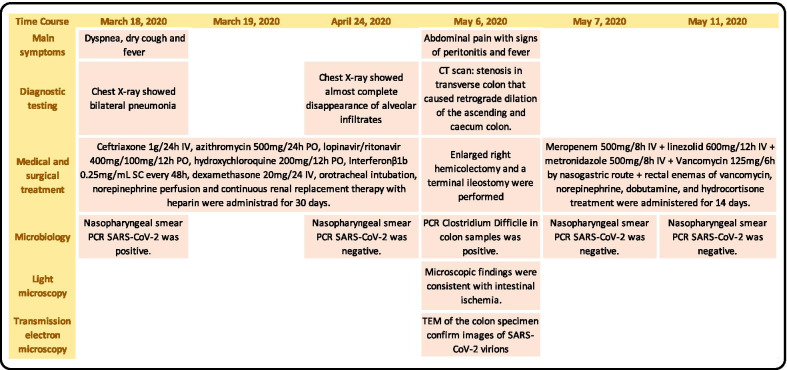
Time course of the patient's 2 (B) main events. This figure shows the main events and complications of the case 2. The first row of the table shows the dates of the events, and the first column describes the complementary examinations carried out

After surgery, the patient was readmitted to the ICU with septic shock and multiorgan failure. Antibiotic treatment was started with wide antibiotic coverage (meropenem 500 mg/8 h IV + linezolid 600 mg/12 h IV + metronidazole 500 mg/8 h IV + Vancomycin 125 mg/6 h via nasogastric route + rectal vancomycin enemas). Norepinephrine, dobutamine, and hydrocortisone treatment were administered for 96 h. Finally, the patient was extubated, and then transferred to a conventional ward to continue rehabilitation until June 4, 2020, when he was discharged from hospital. The final diagnoses were bilateral pneumonia secondary to COVID-19 and colonic ischaemia with endothelial damage related to SARS-CoV-2 (Fig. 6).

## Discussion and conclusion

We present the first two cases in the medical literature of severe intestinal complications (intestinal lymphoma and ischaemic colitis) in which the presence of SARS-CoV-2 was confirmed by TEM.

Case 1 had predominantly digestive manifestations and CT scan was suggestive of a neoplastic process. However, the initial negativity of biopsies for malignancy and the dramatic and rapid reduction of the inflammatory mass, including the size of lymph nodes, in parallel to COVID-19 resolution, suggested an aetiological role of SARS-CoV-2 in the intestinal inflammatory process.

As it is known, corticosteroids are very commonly used in the treatment of COVID-19 cytokine storm and they may also reduce the lymphoma size. But these drugs were not administered to our patient. Initial hypothesis is that SARS-CoV-2 was a colonizer of the intestinal mucosa but the aetiological role of SARS-CoV-2 in the intestinal inflammatory process was suggested by the presence of viral particles with the characteristic features of coronavirus in the cytoplasm of endothelial cells from blood vessels in damaged mucosa. Supporting this view, it is known that SARS-CoV-2 may cause endothelial dysfunction and endotheliitis [12–16]. In addition, macrophages infiltrating the colonic mucosa, identified in our case by light microscopy, could be responsible for the marked fibrotic reaction leading to obstructive symptoms that appeared early during the follow-up. In this sense, macrophages enriched in genes that promote fibrosis and tissue repair have been identified in the lung of patients with severe fibrotic complications from COVID-19 [17].

The diagnosis of DLBCL was finally confirmed in the resected ileocecal specimen. SARS-CoV-2 invasion of the ileocecal mucosa could have induced a local hyperinflammatory response. It was suspected that SARS-CoV-2 had had an oncogenic role, acting as a trigger, as occurs with Epstein-Barr virus and other viruses and with other types of neoplasms (human herpes virus 8 in Kaposi's sarcoma and human T-cell lymphotropic virus in T-cell leukaemia/lymphoma) [18]. To confirm or rule out this hypothesis, RNA*-*ISH of the DLBCL and endothelial cells with SARS-CoV-2 probes was performed with negative results, excluding an oncogenic role in this case.

It is also noteworthy the persistence of SARS-CoV-2 particles in the resection specimen 6 months after COVID-19 recovery and nasopharyngeal SARS-CoV-2 clearance, raising the possibility of latent SARS-CoV-2 infection as it occurs with CMV.

Case 2, in contrast to case 1, had a classical severe COVID-19 with predominantly respiratory symptoms, requiring ICU support. Severe colonic inflammation with perforation was a late complication appearing almost 2 months after admission. It was initially interpreted in the setting of intestinal damage observed in a critically ill patient. The aetiology of this entity is diverse and includes sepsis and pseudomembranous colitis, as well as systemic or local hypoperfusion [19]. Most of these situations concurred with our patient’s condition. With respect to positive PCR for *CD* in a colonic sample, this was considered a contamination [20] but not a true infection, because the patient had no diarrhoea at the time of colonic perforation. In spite of this, the patient’s status was severe enough to warrant wide antibiotic coverage, including specific treatment for *CD.* The presence of SARS-CoV-2 in endothelial cells and blood vessels of the bowel wall gives support to the idea that this virus was one of the main aetiological factors for the ischaemic colitis. SARS-CoV-2 may well act like other viral infections such as CMV, giving rise to inflammatory and ischaemic lesions of the intestine [21, 22]. In addition, CMV has been recognized as one of the significant risk factors for *CD* colonization [23]. Both agents can cause toxic megacolon and bowel perforation, leading to a poor prognosis, as was probably the case with SARS-CoV-2.

With regard to the TEM images, the spikes of the virus particles do resemble ribosomes in tangentially sectioned rough endoplasmic reticulum, but ribosomes are much more electron-dense, rounded, and occasionally bilobated, so at very high magnification, the two ribosomal subunits can be distinguished [11, 24]. Another potential pitfall, particularly in endothelial cells, is pinocytotic vesicles and clathrin-coated exocytotic vesicles. In contrast to coronavirus particles, pinocytotic vesicles have a smooth contour and are often arranged in clusters. Clathrin, on the other hand, does not produce prominent spikes but rather mild irregularities in the surface of the coated vesicle. Thus, careful consideration of this differential allows for very high sensitivity and specificity of electron microscopy in this setting.

The main strengths of this study are firstly the detailed description of SARS-CoV-2 intestinal location by TEM with special emphasis in the differential diagnosis with other cell components such as ribosomes, vesicles and multivesicular bodies which are lookalikes of genuine coronaviruses. In this sense, the expertise of electron microscopists is essential identifying potential ‘decoys’ when looking for SARS-CoV-2 [11]; secondly, the demonstration of SARS-CoV-2 persistence but with a decreasing number in endothelial cells in sequential samples taken within the 6 months after COVID-19 infection, that suggest a latent status. It is also noteworthy the presence of SARS-CoV-2 in endothelial cells instead of in enterocytes giving support to the concept that intestinal damage may be a consequence of blood dissemination, and not through direct enterocyte invasion [11]; It seems to be particularly true in the patient of ischaemic colitis developing a perforation as a late complication 1 month after a severe pneumoniae.

By contrast there are some limitations such as the lack of SARS-CoV-2 assessment in the stools of the patients at admission and during the follow-up and the lack of other morphological and complementary in-situ techniques demonstrating viral components in the intestine (PCR performed on FFPE tissue to detect SARS-CoV-2 was negative in case 1).

This last point is a strong limitation of the study, the reason why the authors want to be very cautious with the interpretation of the reported findings. Molecular detection of SARS-CoV-2 infection in FFPE samples is possible but it is known that tissue processing clearly impacts the accuracy of PCR analysis [25–27]. First, performing PCR on FFPE samples shows lower diagnostic performance compared to fresh samples. This may be due to either limited test sensitivity or sampling problem. There is a possibility of RNA degradation, which is common in clinical samples, particularly when specimens are not immediately stored in a transfer medium suitable for preserving RNA [26, 27]. Second, in summer 2020, time of sample collection, rather than working up a protocol optimized for FFPE tissue, a PCR kit designed for pharyngeal swabs was used instead. However, detection of viral RNA and proteins of the virus does not necessarily reflect the presence of intact and infectious particles whereas TEM is able to demonstrate assembled virions in COVID-19 infected patients. We are aware that causality cannot be demonstrated only with two cases, but the presence of assembled virions of SARS-CoV-2 in the endothelial cells of the damaged tissue, confirmed on repeated samples over time suggests that could have an aetiological role, despite not having been confirmed by other methods.

In conclusion, severe intestinal complications are infrequent in COVID-19 patients. The cases reported herein demonstrated for the first time by TEM the presence of assembled virions of SARS-CoV-2 in endothelial cells of the intestinal mucosa and persistence 6 months after COVID-19 acquisition and resolution. A pathophysiological role of SARS-CoV-2 in intestinal damage is suggested as well as persistence of latent infection. Negative ISH of the DLBCL cells with SARS-CoV-2 probes argues against an oncogenic role of SARS-CoV-2.

## Data Availability

The authors declare that all other relevant data generated or analysed during this study are included in the article, the extended data file, or the supplementary information files. Materials, protocols and images are available from the corresponding author on reasonable request.

## Abbreviations

COVID-19: Coronavirus disease 2019
ACE2: Angiotensin-converting enzyme 2
TEM: Transmission electron microscopy
CRP: C-reactive protein
INR: International normalized ratio
CT: Computed tomography
PET: Positron emission tomography
SUV: Standard uptake value
PCR: Polymerase chain reaction
ISH: In situ Hybridization
CMV: Cytomegalovirus
FFPE: Formalin-fixed/paraffin-embedded
DLBCL: Diffuse large B-cell lymphoma
ICU: Intensive care unit
CD: *Clostridium difficile*
